# KRAS–SOS-1 Inhibition as New Pharmacological Target to Counteract Anaplastic Thyroid Carcinoma (ATC)

**DOI:** 10.3390/ijms26062579

**Published:** 2025-03-13

**Authors:** Deborah Mannino, Rossella Basilotta, Fabiola De Luca, Giovanna Casili, Emanuela Esposito, Irene Paterniti

**Affiliations:** Department of Chemical, Biological, Pharmaceutical and Environmental Sciences, University of Messina, Viale Ferdinando Stagno D’Alcontres 31, 98166 Messina, Italy; deborah.mannino@unime.it (D.M.); rossella.basilotta@unime.it (R.B.); fabiola.deluca@unime.it (F.D.L.); gcasili@unime.it (G.C.); eesposito@unime.it (E.E.)

**Keywords:** anaplastic thyroid carcinoma, KRAS, son of sevenless 1, BAY-293

## Abstract

Anaplastic thyroid carcinoma (ATC) is the most aggressive type of thyroid cancer. Tumor cells have been shown to activate alternative signaling pathways, making treatments less effective. One of the major proteins involved in the progression of ATC is the proto-oncogene KRAS that belongs to a group of small guanosine triphosphate (GTP)-binding proteins. Despite its recognized importance in cancer malignancy, KRAS is considered non-druggable and has never been studied in the field of ATC. In this context, a new synthetic molecule, BAY-293, has recently been developed that selectively inhibits the KRAS–SOS-1 interaction. Based on these findings, the aim of this study was to evaluate for the first time the antitumor effect of BAY-293 using in vitro and in vivo models of ATC. The in vitro model included different thyroid cancer (TC) cell lines used to study the effect of BAY-293 on the modulation of mitogen-activated protein kinase (MAPK) pathways, apoptosis, and cell migration. To confirm the in vitro findings and better mimic the complex tumor microenvironment, an in vivo orthotopic model of ATC was used. The results of the study indicate that BAY-293, both in vitro and in vivo, effectively blocked the KRAS/MAPK/ERK pathway and β-catenin, which act as downstream effectors essential for cell migration, and increased the apoptotic process by slowing the progression of ATC. In conclusion, this study demonstrated that KRAS/SOS-1 inhibition could be a promising therapeutic target for the treatment of ATC and highlighted BAY-293 as an innovative molecule that needs further research to fully evaluate its efficacy in the field of thyroid cancer.

## 1. Introduction

Anaplastic thyroid carcinoma (ATC) is a highly aggressive thyroid tumor composed of undifferentiated follicular cells [1]. This rare type of thyroid carcinoma (1–2% of all thyroid malignancies) usually develops in elderly patients and presents as a rapidly growing, firm, and infiltrative neck mass [2]. The prognosis of ATC is severe with a median survival period of less than 6 months and a mortality rate of more than 90% [3]. Nuclear pleomorphism, tumor necrosis, increased mitosis, and infiltrative growth are key features for a conclusive diagnosis of ATC, and immunohistochemistry for Ki-67 is useful to confirm ATC and other high-risk thyroid carcinomas [4]. Therapeutic approaches for anaplastic thyroid cancer are very limited. ATC often results in poor surgical resectability, and radioactive iodine treatment is generally ineffective [5]. Thyroid cancer guidelines therefore recommend the use of target therapy [6]. Hence, the therapy used depends on the results of the molecular test and the mutations involved. Other recommended regimens include treatment with anthracyclines and taxanes, which are generally not very effective for advanced anaplastic disease. However, in some cases, these drugs may contribute to disease response or maintenance of stable disease [7].

The undifferentiated subtype of thyroid cancer, ATC, results from the gradual dedifferentiation of pre-existing well-differentiated thyroid tumors, such as papillary thyroid carcinoma (PTC) and follicular thyroid carcinoma (FTC). These processes can be driven and amplified by hyperactivation of the rat sarcoma virus (RAS) oncogene [8]. Several studies observed that FTCs and PTCs with focal areas of poorly differentiated histology are often positive for the RAS mutation [9,10]. RAS is a family of GTP-binding proteins located upstream of BRAF that acts through MAPK signaling pathways. *HRAS*, *KRAS*, and *NRAS* encode four different but related proteins (HRAS, NRAS, KRAS4A, and KRAS4B) that are cardinal in the control of cell growth, differentiation, and survival [11]. In particular, the KRAS protein transduces extracellular signals through cell surface receptors from the inactive state [bound to guanosine diphosphate (GDP)] to the active state [bound to guanosine triphosphate (GTP)]. KRAS protein activation orchestrates intracellular signaling cascades that regulate tumor cell survival and proliferation [12]. Aberrant activation of KRAS occurs in approximately 1 in 7 of all human tumors, making it the most frequently mutated oncogene [13]. This aberration can be caused by deregulated upstream signaling, loss of GTPase-activating protein function, or oncogenic mutations that result in increased GTP-bound KRAS and persistent downstream signaling [14].

Significant efforts and progress have recently been made in the development of novel therapies that directly inhibit KRAS or target functionally important RTK/RAS effector pathways, such as the mitogen-activated protein kinase (MAPK) or phosphoinositide 3-kinase (PI3K) pathways. However, in most cases, the development of acquired resistance is inevitable, and the toxicity of combination therapies may become prohibitive to tolerate [15]. This requires the development of new strategies to overcome these clinical dilemmas. An attractive approach to target KRAS and its interaction with GTP cargo could be through the son of sevenless 1 (SOS-1) protein [16]. SOS-1, also known as guanine nucleotide exchange factor (GEF), increases GTP turnover and regulates the fraction of KRAS in the active state and cell proliferation. Furthermore, SOS-1 not only promotes the production of active GTP-bound KRAS at the catalytic site, but also enhances its GEF function allosterically. Therefore, given its direct protein–protein interaction with KRAS, targeting SOS-1 may have advantages over other indirect approaches for suppressing KRAS signaling. In addition, targeting the SOS-1-KRAS interaction, due to the functional compensation of its paralog SOS-2 and the lack of requirement in normal versus tumor cells, might also be less toxic [17].

Several small molecule inhibitors, such as BAY-293, have been developed to abolish GTP recharging and to impair the interaction between SOS-1 and KRAS, resulting in antiproliferative activity [18]. BAY-293 is a 6,7-dimethoxy-2-methyl-N-[(1R)-1-[4-[2-(methylaminomethyl) phenyl]thiophen-2-yl]ethyl]quinazoline-4-amine with a molecular weight of 448.6 g/mol. It is a selective and potent inhibitor of the KRAS–SOS-1 interaction with an IC50 of 21 nM. BAY-293 was shown to inhibit KRAS activation in HeLa cells, with IC50 values in the submicromolar range, and showed efficient antiproliferative activity against wild-type KRAS cell lines (K-562, MOLM-13) and *KRASG12C* mutant cell lines (NCI-H358, Calu-1) [18]. Furthermore, an in vitro study on pancreatic cancer cells demonstrated that BAY-293 effectively inhibited cell proliferation by blocking KRAS activation and reducing the phosphorylation of downstream effectors such as MAPKs [19,20]. In particular, BAY-293 is attractive for its pan-RAS inhibition independently of the type of KRAS mutations [19]. However, its antitumor effect in the field of thyroid cancer has never been investigated before.

Based on this evidence, the purpose of this investigation was to study the efficacy of the pan-KRAS inhibitor BAY-293 for the treatment of ATC.

## 2. Results

### 2.1. In Vitro Results

#### 2.1.1. The Effect of BAY-293 on Reducing Thyroid Cancer (TC) Cell Viability

**Figure 1:**
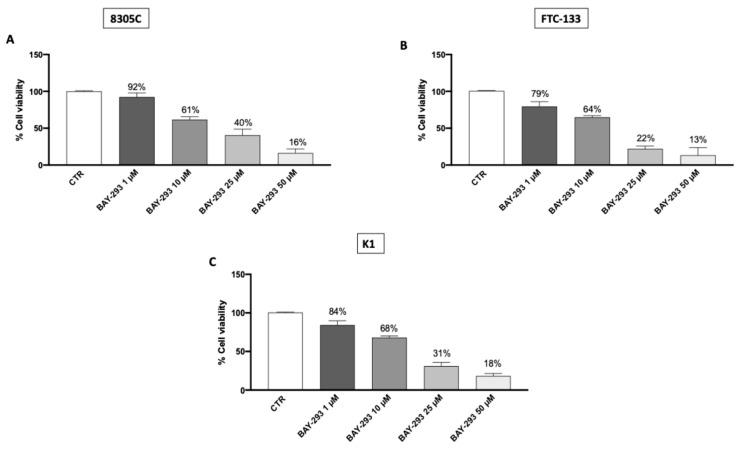
Effect of BAY-293 on cell viability. BAY-293 treatment was able to significantly reduce cell viability in a concentration-dependent manner compared to the control group (CTR) in the anaplastic thyroid cancer cell line 8305C (**A**), the follicular thyroid cancer cell line FTC-133 (**B**), and the papillary thyroid cancer cell line K1 (**C**). Data are representative of at least three independent experiments. Values are means ± SDs. We used one-way ANOVA tests followed by Bonferroni post hoc tests for multiple comparisons.

The MTT assay was performed to evaluate the viability of 8305C, FTC-133, and K1 cells after 24 h treatment with BAY-293. Our findings demonstrated that BAY-293 treatments were able to reduce cell viability in a concentration-dependent way (Figure 1A–C). Based on these results we chose to perform additional investigations focusing just on BAY-293 at concentrations of 1 μM, 10 μM, and 25 μM, as these were found to be the lowest concentrations with the greatest cytotoxic effects. Furthermore, our results demonstrated that BAY-293 had significant cytotoxic efficacy in all three TC cell lines. However, we chose to focus exclusively on the 8305C cell line, since anaplastic carcinoma represents the most aggressive form of TC. Furthermore, since our objective also included investigating the effect of BAY-293 in an in vivo mouse model and given that the literature indicates that the 8305C cell line is commonly used in experimental models of orthotopic thyroid carcinoma, we chose to use this cell line [21,22].

#### 2.1.2. ATC Cell Migration and Proliferation Were Reduced by BAY-293 Treatment

**Figure 2:**
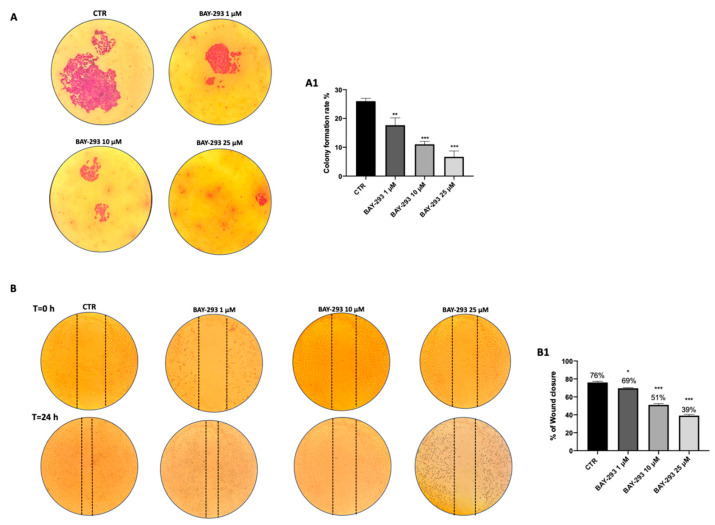
Effect of BAY-293 on colony formation and migration. The colony formation assay using 8305C cells treated with BAY-293 for 24 h at concentrations of 1 μM, 10 μM, and 25 μM showed a significant reduction in colony formation compared to control untreated 8305C cells (CTR) ((**A**), see score panel (**A1**)). ** *p* < 0.01 vs. CTR; *** *p* < 0.001 vs. CTR. The wound healing assay (scratch test) revealed a significant reduction in the number of cells migrating to the scratched area and, thus, a reduced percentage of wound closure following 24 h of BAY-293 treatment at concentrations of 1 μM, 10 μM, and 25 μM ((**B**), see score panel (**B1**)). * *p* < 0.05 vs. CTR; *** *p* < 0.001 vs. CTR. Data are representative of at least three independent experiments. Values are means  ±  SDs. We used one-way ANOVA tests followed by Bonferroni post hoc tests for multiple comparisons.

By preventing the transition from inactive GDP to active GTP, BAY-293 alters the interaction between SOS-1 and KRAS, resulting in antiproliferative activity [23]. BAY-293’s impact on 8305C cell migration was assessed using an in vitro wound healing assay. After a scratch was generated, confluent cells were treated with BAY-293 for 24 h. Our findings demonstrated that at all the concentrations tested, BAY-293 significantly decreased the number of cells moving to the scratched area (Figure 2A, see colony formation rate in panel A1). In addition, we assessed the ability of 8305C cells to form colonies after treatment with BAY-293 at concentrations of 1 μM, 10 μM, and 25 μM. According to the 0.1% (*w*/*v*) crystal violet staining results, BAY-293 considerably reduced colony development of 8305C cells at all the concentrations (Figure 2B, see % of wound closure in panel B1).

#### 2.1.3. The Impact of BAY-293 on Modulation of the KRAS/SOS-1 Pathway

**Figure 3:**
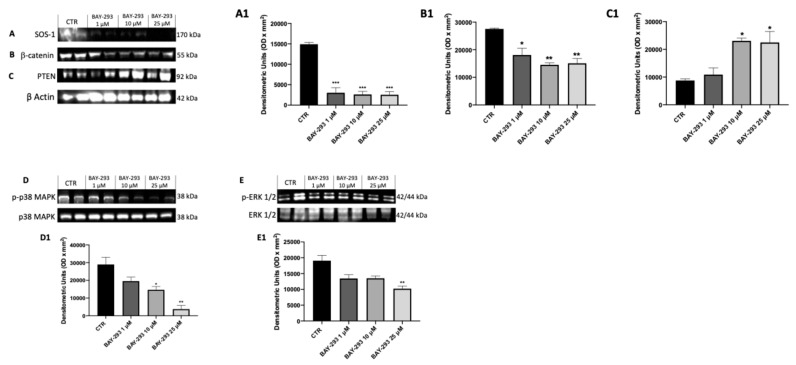
Effect of BAY-293 on the KRAS/SOS-1 pathway in 8305C cells. The blots revealed a significant modulation of KRAS/SOS-1 marker expression following BAY-293 treatment. BAY-293 at concentrations of 1 μM, 10 μM, and 25 μM was able to significantly reduce the expression of son of sevenless 1 (SOS-1) (**A**), see densitometric analysis panel (**A1**) and β-catenin (**B**), see densitometric analysis panel (**B1**) compared to untreated 8305C cells. At concentrations of 10 μM and 25 μM, BAY-293 significantly reduced phosphorylated p38 mitogen-activated protein kinase (p38 MAPK) (**D**), see densitometric analysis panel (**D1**) levels compared to untreated cells, while only the highest concentrations of 25 μM was able to significantly reduce extracellular signal-regulated kinase (p-ERK) levels (**E**), see densitometric analysis panel (**E1**). Treatment with BAY-293 (10 μM and 25 μM) was also able to increase the expression of tumor suppressor protein phosphatase and tensin homolog (PTEN) (**C**), see densitometric analysis panel (**C1**). *** *p* < 0.001 vs. control (CTR); ** *p* < 0.01 vs. CTR; * *p* < 0.05. Our data are the result of three experimental replicates. Data are representative of at least three independent experiments. Values are means ± SDs. We used one-way ANOVA tests followed by Bonferroni post hoc tests for multiple comparisons.

BAY-293 has been extensively demonstrated to influence multiple molecular mechanisms, such as the mitogen-activated protein kinase (MAPK) signaling pathway and apoptosis, providing further insight into its antitumor activity [24]. To confirm the action on the target, we used western blot analysis to evaluate the expression of KRAS/SOS-1 pathway-related proteins [SOS-1, phosphatase and tensin homolog (PTEN), phosphorylated p38 mitogen-activated protein kinase (p38), extracellular signal-regulated kinase (ERK), β-catenin]. Figure 3 displays a considerable reduction in SOS-1 after BAY-293 treatment compared to untreated cells, confirming the inhibition of the target (Figure 3A, panel A1). SOS-1 inhibition results in a high reduction in p-ERK activity and blockade of the RAS/MAP kinase pathway. Indeed, the SOS-1/K-RAS/MEK-ERK signaling pathway is responsible for regulating cell proliferation, differentiation, and survival of thyroid cancer cells [25]. Furthermore, it has recently been demonstrated that hyperactivation of the KRAS/SOS-1 signal via a positive feedback loop through the MEK–ERK pathway leads to the accumulation of β-catenin, which contributes to tumorigenesis and malignant transformation [26]. Our results showed a significant downregulation of p-p38 MAPK following treatment with BAY-293 at concentrations of 10 μM and 25 μM (Figure 3D,D1) and reduced levels of p-ERK1 following treatment with BAY-293 at the concentration of 25 μM (Figure 3E,E1). Moreover, we observed a significant reduction in β-catenin levels in 8305C cells treated with BAY-293 at concentrations of 1 μM, 10 μM, and 25 μM (Figure 3B,B1), suggesting a role in the inhibition of the RAS/MAP kinase pathway. Simultaneously, our data showed that BAY-293 treatment results in an upregulation of the tumor suppressor protein PTEN, particularly at the higher concentrations of 10 and 25 μM (Figure 3C,C1).

#### 2.1.4. BAY-293 Activated ATC Cell Apoptosis

**Figure 4:**
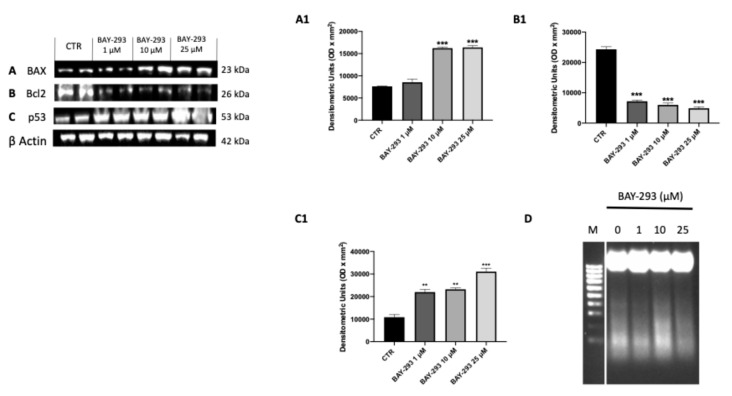
Effect of BAY-293 on apoptosis marker expression in 8305C cells. Western blot analysis revealed a significant modulation of apoptosis markers expression following BAY-293 treatment. BAY-293 was able to significantly increase the levels of pro-apoptotic markers Bcl2-associated X, apoptosis regulator (BAX) (**A**), see densitometric analysis panel (**A1**) and tumor protein (p53) ((**C**), see densitometric analysis panel (**C1**) and significantly reduced the expression of anti-apoptotic protein B-cell leukemia/lymphoma 2 protein (Bcl2) (**B**), see densitometric analysis panel (**B1**). Analysis of genomic DNA fragmentation in 8305C cells after treatment with BAY-293 at concentrations of 1 μM, 10 μM, and 25 μM for 24 h revealed a typical ladder pattern of internucleosomal fragmentation in cells after 10 μM BAY-293 treatment for 24 h (**D**). *** *p* < 0.001 vs. CTR; ** *p* < 0.01 vs. CTR. Data are representative of at least three independent experiments. Values are means  ±  SDs. We used one-way ANOVA tests followed by Bonferroni post hoc tests for multiple comparisons.

SOS-1 promotes cell survival through the activation of the RAS–MAPK and PI3K–AKT pathways, enhancing the expression of anti-apoptotic proteins and inhibiting pro-apoptotic factors [27]. Treatment with BAY-293 revealed that SOS-1 inhibition can lead to reduce cell proliferation by promoting apoptosis in ATC cells. The effect of BAY-293 treatment on the expression of apoptotic markers Bcl2-associated X, apoptosis regulator (BAX); tumor protein (p53); and B-cell leukemia/lymphoma 2 protein (Bcl2) was assessed by Western blot analysis. According to our findings, BAY-293 was able to significantly increase the levels of pro-apoptotic proteins BAX and p53 and to decrease the expression of the anti-apoptotic Bcl2 protein compared to the control cells (Figure 4A–C,A1–C1). The induction of the apoptotic process was confirmed using the DNA fragmentation assay. Following agarose gel electrophoresis of 8305C cells treated with 10 μM BAY-293 for 24 h, a typical ladder pattern of internucleosomal fragmentation was observed (Figure 4D).

### 2.2. In Vivo Results

#### 2.2.1. BAY-293 Improved Histopathological Features in an ATC Orthotopic Model

**Figure 5:**
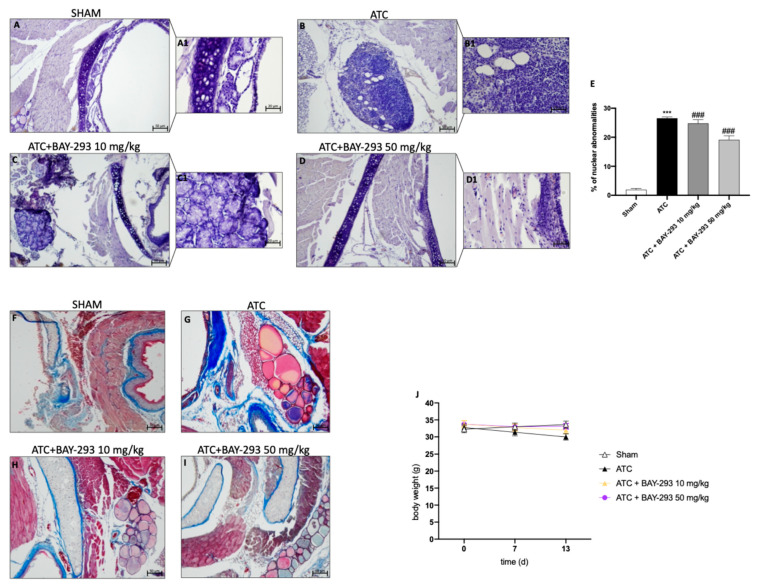
Effect of BAY-293 on tumor growth in an orthotopic model of ATC. Hematoxylin and eosin staining revealed that BAY-293 treatment at doses of 10 mg/kg (**C**,**C1**) and 50 mg/kg (**D**,**D1**) was able to reduce features of a high-grade malignant neoplasm such as high-grade nuclear atypia, marked cellular pleomorphism, necrosis, and neutrophil infiltration compared to the ATC mice (**B**,**B1**), see score panel (**E**); (**A**,**A1**) Sham group. Masson’s trichrome staining of ATC tumors. The collagen is stained in blue and cytoplasm is stained in red. BAY-293 treatment at doses of 10 and 50 mg/kg reduced infiltration of collagen fibers around the tumors compared to the ATC group (**F**–**I**). No important changes in the animals’ body weights were shown during the experiments (**J**). Values are means ± SDs. Distribution of values come from individual animals. One-way ANOVA test. *** *p* < 0.001 vs. sham; ### *p* < 0.001 vs. ATC. The images are shown at 20× (50 μm of the bar scale) and 40× magnification (20 μm of the bar scale).

The histological evaluation of the ATC group (Figure 5B,B1 score panel E) revealed characteristics of a high-grade malignant tumor, including necrosis, significant cellular pleomorphism, high-grade nuclear atypia, and extensive neutrophilic inflammation compared to sham group (Figure 5A,A1 score panel E) [23]. As shown in Figure 5, our findings revealed that BAY-293 treatment at doses of 10 and 50 mg/kg was able to improve these pathological characteristics (Figure 5C,D,C1,D1, score panel E). In addition, malignant tumor cells can induce the formation of a supporting stroma, consisting of collagen type I and III fibers, which evolve into fibrosis in most solid tumors [28]. Masson’s trichrome-stained histological sections were performed to assess the extent of fibrosis in 8305C orthotopic tumors. Comparing the ATC group (Figure 5G) with the sham group (Figure 5F), it is evident that the ATC group shows greater infiltration of collagen fibers around the tumors. According to our data, these fibrotic characteristics could be reduced by BAY-293 treatment at doses of 10 and 50 mg/kg, as shown in Figure 5H,I. Moreover, our results demonstrated that no significant changes in body weight were reported in all mice during the experiment (from day 0 to day 13) (Figure 5J).

#### 2.2.2. BAY-293 Reduced Ki-67 Expression and Increased Apoptosis in the Orthotopic Model

**Figure 6:**
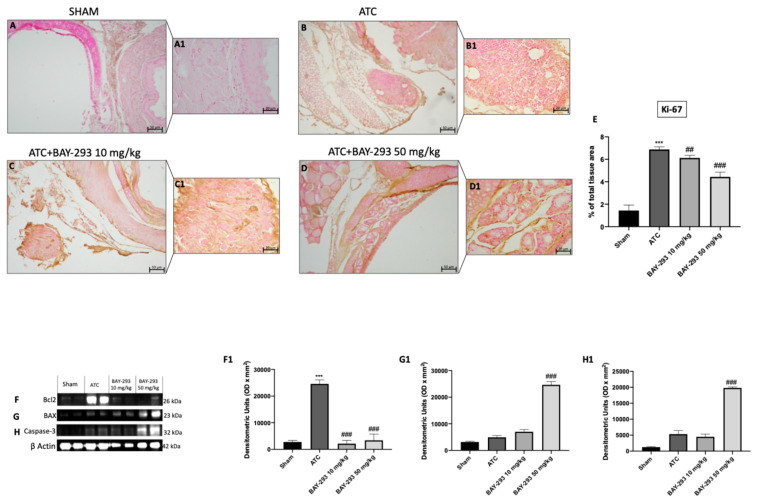
Effects of BAY-293 on the cell proliferation marker Ki-67 and the apoptosis pathway. Immunohistochemical analysis of Ki-67 demonstrated that ATC tissues (**B**,**B1**), see score panel (**E**) showed a high number of Ki-67-positive cells compared to tissues from the sham group (**A**,**A1**), see score panel (**E**). BAY-293 treatment at doses of 10 mg/kg (**C**,**C1**), see score panel (**E**) and 50 mg/kg (**D**,**D1**), see score panel (**E**) significantly reduced the number of Ki-67-positive cells compared to the ATC group. Western blot analysis conducted on thyroid tissue lysates demonstrated that BAY-293 reduced anti-apoptotic protein B-cell leukemia/lymphoma 2 protein (Bcl2) (**F**), score (**F1**) and increased pro-apoptotic protein Bcl2-associated X, apoptosis regulator (BAX) (**G**), score (**G1**) and caspase-3 (**H**), score (**H1**). Values are means ± SDs. Distribution of values come from individual animals. One-way ANOVA test. *** *p* < 0.001 vs. sham; ## *p* < 0.01 vs. ATC; ### *p* < 0.001 vs. ATC. The images are shown at 20× (50 μm of the bar scale) and 40× magnification (20 μm of the bar scale).

Among the histopathological features of ATC, beyond necrosis, extensive neutrophilic inflammation, and the presence of fibrous tissue, it is characterized by a high Ki-67 proliferation index based on the percentage of positive tumor nuclei (>30%) [29]. Ki-67 overexpression correlates with adverse outcomes in undifferentiated thyroid cancer [30]. According to our findings, the ATC group (Figure 6B,B1, see score E) compared to the sham group (Figure 6A,A1, see score E) showed a significant overexpression of Ki-67-positive nuclei. Figure 6 illustrates how BAY-293 treatment at doses of 10 and 50 mg/kg significantly reduced the expression Ki-67 in orthotopic tumors, thereby contributing to the reduction of tumoral proliferation and invasion (Figure 6C,C1,D,D1, see score panel E). Moreover, Western blot results conducted on samples collected in the orthotopic model confirmed that BAY-293 was able to induce activation of programmed cell death. Treatment with BAY-293 led to a downregulation of the antiapoptotic marker Bcl2 (Figure 6F,F1) and to an increase in the expression levels of the pro-apoptotic proteins BAX (Figure 6G,G1) and caspase 3 (Figure 6H,H1), confirming the results obtained in vitro.

#### 2.2.3. BAY-293 Inhibits the KRAS/SOS-1 Pathway in the Orthotopic Model

**Figure 7:**
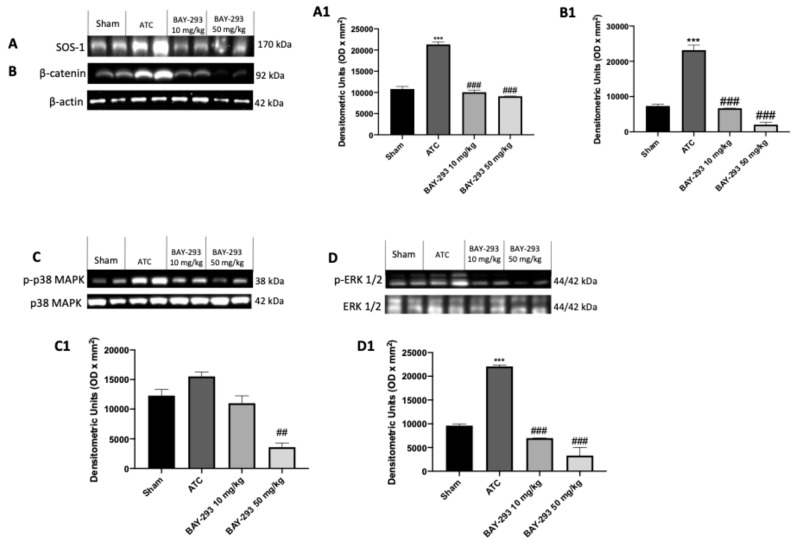
Effect of BAY-293 on the KRAS/SOS-1 pathway in an orthotopic model of ATC. The blots revealed a significant reduction in son of sevenless 1 (SOS-1) after treatment with BAY-293 at the doses of 10 mg/kg and 50 mg/kg compared to sham mice (**A**), densitometric analysis panel (**A1**). BAY-293 significantly reduced β-catenin levels (**B**), densitometric analysis panel (**B1**) and phosphorylated extracellular signal-regulated kinase (p-ERK) levels (**D**), densitometric analysis panel (**D1**) at doses of 10 mg/kg and 50 mg/kg compared to ATC mice. Only the highest dose of 50 mg/kg of BAY-293 was able to significantly reduce the expression of phosphorylated p38 mitogen-activated protein kinase (p38 MAPK) (**C**), densitometric analysis panel (**C1**) compared to the ATC group. Values are means ± SDs. Distribution of values come from individual animals. One-way ANOVA test. *** *p* < 0.001 vs. sham; ## *p* < 0.01 vs. ATC; ### *p* < 0.001 vs. ATC.

To confirm the action on the target and the consequent modulation of the downstream factors of the KRAS/SOS-1 pathway, we also performed molecular biology analyses on the thyroid samples collected from the orthotopic model. Our results showed elevated levels of SOS-1 and p-ERK in the ATC group compared to the sham group (Figure 7A,A1,D,D1). However, treatment with BAY-293 at both doses of 10 mg/kg and 50 mg/kg was able to downregulate SOS-1 and p-ERK (Figure 7A,A1,D,D1). Furthermore, the higher dose of BAY-293 (50 mg/kg) significantly reduced p-p38 levels compared to the ATC group (Figure 7C,C1). Furthermore, the expression of β-catenin was significantly elevated in the ATC group compared to the sham group (Figure 7B,B1). Otherwise, β-catenin levels were significantly reduced following treatment with BAY-293 at doses of 10 and 50 mg/kg compared to the ATC group (Figure 7B,B1).

## 3. Discussion

ATC is a rare and aggressive cancer that is often diagnosed late and characterized by a poor prognosis, leading to 90% of cases to death within one year of diagnosis [31]. The management of ATC involves a multimodal therapeutic approach that combines surgical resection and chemoradiotherapy [32]. However, the efficacy of the current therapeutic approach is limited. Hence, the identification of usable therapeutic targets is essential to improve clinical outcomes. In this context, targeted therapy against protein tyrosine kinases has shown promising results in preclinical and clinical studies [33]. RAS mutations are among the main drivers of poorly differentiated carcinomas and occurring in 29% of ATCs [34]. Molecular genetic studies suggest that RAS oncogenes are frequently mutated in ATC as a result of progression from RAS-mutated FTCs [35,36]. In this aggressive form of thyroid cancer, mutations in KRAS can lead to constitutive activation of the RAS/MAPK pathway [37]. Overactive SOS-1 can further enhance this pathway, leading to increased tumor growth and resistance to apoptosis. In this study, we used a potent blocker of the interaction between KRAS and SOS-1, namely, BAY-293, to determine, for the first time, whether pan-KRAS inhibition could influence the proliferation and migration of ATC cells. BAY-293, through interactions with its target and effectors, was able to disrupt downstream signaling pathways involved in cancer cell proliferation, like the MAPK/ERK pathway. These pathways are often involved in promoting cell growth, survival, and proliferation in cancer cells [38]. In particular, following the KRAS/SOS-1 interaction, activation and dimerization of RAF, a member of a family of serine/threonine (Ser/Thr) kinases, occurs. Subsequently, RAF stimulates mitogen-activated protein kinases 1 and 2 (MEK1 and MEK2), which in turn activate downstream extracellular signal-regulated kinases 1 and 2 (ERK1 and ERK2). MEK1 and MEK2 belong to the dual-specificity kinase (DSK) family, responsible for phosphorylating Tyr and Ser/Thr residues within the activation loop of their substrate MAP kinases [39]. Activated MEKs directly interact with ERK, which then translocates to the nucleus, where it phosphorylates and activates various transcriptional factors, allowing cell cycle progression through G0/G1 mitogenic signals [40]. The results of this study demonstrated that BAY-293 has a significant cytotoxic effect on thyroid cancer cell lines, reducing their viability in a concentration-dependent manner. In addition, inhibition of the transition from inactive GDP to active GTP with BAY-293 alters the interaction between SOS-1 and KRAS, a key mechanism for KRAS activation and its ability to promote cell proliferation. We demonstrated that inhibition of this interaction is associated with antiproliferative activity in the context of ATC as observed by the wound healing assay and the colony formation assay results. This is an important result since cell migration is a crucial step in the process of metastasis, and its inhibition could reduce the ability of tumor cells to invade surrounding tissues. Moreover, our results showed a significant downregulation of the RAS/MAP kinase pathway through the modulation of p-ERK, p-p38 MAPK, and β-catenin expression both in vitro and in vivo, suggesting a role in the inhibition of ATC cell proliferation and migration. In addition to reducing proliferation, BAY-293 was able to induce programmed cell death in ATC tumor cells by disrupting survival signals mediated through KRAS. The PI3K/AKT pathway, also regulated by KRAS, promotes cell survival and resistance to apoptosis [41]. Activated PI3K leads to the production of PIP3 and activation of AKT, promoting cell survival and growth. Loss of PTEN function results in unchecked AKT activation. When KRAS is mutated and PTEN is lost or inactivated, there is a synergistic effect, leading to robust activation of the PI3K/AKT pathway [42]. The obtained results describe an interesting mechanism through which BAY-293 exerts potentially promising therapeutic effects in the treatment of tumors. In particular, the inhibition of KRAS activation by BAY-293 led to an increase in PTEN expression. Its activation in this context seems to have a crucial role in inducing apoptotic signals in tumor cells. The effect of BAY-293 in activating the apoptotic process was observed in both in vitro and in vivo studies. Furthermore, DNA fragmentation and caspase 3 activation are classical indicators of apoptosis, suggesting that BAY-293 effectively promotes programmed cell death, thus reducing tumor cell proliferation. Furthermore, the increase in the levels of pro-apoptotic proteins such as BAD, BID, and p53 and the reduction in anti-apoptotic proteins such as Bcl2 suggest that the pathways that promote cell death are enhanced. BAD and BID, for example, are known to promote mitochondrial membrane permeabilization, a critical step in the initiation of apoptosis. p53 is a well-known “guardian of the genome” that stimulates apoptosis in response to DNA damage, while Bcl2 is an anti-apoptotic protein that normally prevents cell death. This suggests that BAY-293 may be able to restore a favorable balance for apoptosis in tumor cells by counteracting the anti-apoptotic defenses commonly activated in tumors. This approach may be particularly advantageous for the treatment of tumors in which KRAS is aberrantly activated, such as ATC. These findings confirmed the action of BAY-293 on the target and are consistent with preclinical studies that have demonstrated the effectiveness of BAY-293 in reducing KRAS signaling and tumor growth in cell lines and animal models [43,44,45]. The impact of BAY-293 on ATC proliferation was observed directly through the reduction in the expression of the proliferation marker Ki-67. Monitoring Ki-67 levels in response to BAY-293 treatment provides a valuable tool for evaluating the efficacy of this inhibitor.

## 4. Materials and Methods

### 4.1. In Vitro Studies

#### 4.1.1. Materials

BAY-293 was provided by the Sigma-Aldrich Company (Sigma-Aldrich, St. Louis, MO, USA, cat. SML2703). Every chemical used was of the best commercial grade possible. Nonpyrogenic saline (0.9% NaCl; Baxter Healthcare Ltd., Thetford, Norfolk, UK) was used to make all stock solutions.

#### 4.1.2. Cell Cultures

Human TC cell lines FTC-133 (follicular thyroid carcinoma cells), K1 (primary papillary TC cells), and 8305C (undifferentiated anaplastic TC cells) were acquired from ATCC (Manassas, VA, USA). The culture media used for the cells was RPMI-1640 (Sigma-Aldrich, cat. R8758), which was enhanced with 10% fetal bovine serum (FBS, Life Technologies, Gibco^®^; Carlsbad, CA, USA), 100 U/mL of penicillin, and 100 μg/mL of streptomycin. Every cell line was maintained in an incubator at a temperature of 37 °C and 5% CO_2_.

#### 4.1.3. Cell Viability (MTT Assay)

3-(4,5-Dimethylthiazol-2-yl)-2,5-diphenyltetrazolium bromide (MTT) (M5655; Sigma-Aldrich), a mitochondria-dependent dye for living cells, was used to assess the cell viability of FTC-133, K1, and 8305C cells. Cells were plated at a density of 4 × 10^4^ cells/well on 96-well plates. Following a 24 h period, BAY-293 was applied to FTC-133, K1, and 8305C cells at progressively higher doses of 1 μM, 10 μM, 25 μM and 50 μM dissolved in dimethyl sulfoxide (DMSO) for a whole day. Following a 24 h incubation period at 37 °C with MTT (0.2 mg/mL) for 1 h, cells were lysed using 100 μL of DMSO, and the optical density at 540 nm (OD540) was measured to determine the degree of reduction of MTT to formazan [46].

#### 4.1.4. Experimental Groups

1. Control group: TC cell lines: FTC-133, K1, and 8305C;

2. BAY-293 1 μM group: FTC-133, K1, and 8305C cells were treated with BAY-293 1 μM for 24 h;

3. BAY-293 10 μM group: FTC-133, K1, and 8305C cells were treated with BAY-293 10 μM for 24 h;

4. BAY-293 25 μM group: FTC-133, K1, and 8305C cells were treated with BAY-293 25 μM for 24 h;

#### 4.1.5. Western Blot Analysis for Cell Lysates

To prepare cell lysates, 8305C cells were twice washed in ice-cold phosphate buffered saline (PBS), collected, and then resuspended in lysis buffer that contained a protease cocktail of inhibitors (Catalog No. 11836153001; Roche, Switzerland), 150 μL of NaCl, 10 mM NaF, and 20 mM Tris-HCl pH 7.5. After 40 min, cell lysates were centrifuged at 1529× *g* for 15 min at 4 °C. Using bovine serum albumin as a standard, the Bio-Rad protein assay (Bio-Rad Laboratories, Hercules, CA, USA) was used to quantify the protein concentration. Following a 5-min heating period to 95 °C, the samples were separated into equal volumes of proteins using 10–15% sodium dodecyl sulfate-polyacrylamide gel electrophoresis (SDS-PAGE) and then transferred to a PVDF membrane (Immobilon-P, catalog # 88018; Thermo Fisher Scientific, Waltham, MA, USA). Anti-PTEN (1:500; sc-7974; Santa Cruz Biotechnology, Dallas, TX, USA), anti-SOS-1 (1:500; sc-17793; Santa Cruz Biotechnology), anti-p-p38 MAPK (1:500; sc-166182, Santa Cruz Biotechnology), anti-p-ERK (1:500; sc-7383; Santa Cruz Biotechnology), p38 MAPK (1:500; sc-7972; Santa Cruz Biotechnology), ERK (1:500; sc-514302; Santa Cruz Biotechnology), β-catenin (1: 500; sc-7963; Santa Cruz Biotechnology), anti-BID (1:500; sc-11423; Santa Cruz Biotechnology), anti-BAX (1:500; sc-20067; Santa Cruz Biotechnology), and anti-p53 (1:500; sc-126; Santa Cruz Biotechnology,) were the primary antibodies utilized. Dilutions of the antibodies were prepared in PBS, 5% *w*/*v* milk powder, and 0.1% Tween-20 (PMT). The membranes were incubated at 4 °C for a whole night. After that, the membranes were treated for 1 h at room temperature with a secondary antibody (1:2000; Jackson ImmunoResearch, West Grove, PA, USA). Additionally, we used β-actin antibody (cytosolic fraction 1:500; sc-47778; Santa Cruz Biotechnology) to guarantee that equivalent amounts of protein lysate have been loaded. An enhanced chemiluminescence (ECL) detection system mixture (Thermo Fisher Scientific) was used to detect the signals.

#### 4.1.6. Wound Healing Assay (Scratch Test)

To obtain a confluent monolayer, 2 × 10^6^ 8305C cells were plated on 60 mm plates (Corning Cell Culture, Tewksuby, MA, USA). A straight line was created by scratching the cell monolayer 24 h later with a P200 pipette tip. Following the removal of debris from each plate, cells were subjected to 24 h of treatment with BAY-293 at increasing doses (1 μM, 10 μM, and 25 μM). In contrast, standard culture media was employed in the control group. In order to document the extent of the wound and, consequently, the cell migration capacity, phase contrast microscopy images of every plate were taken at 0 and 24 h. Image J 1.53a software was used to calculate the cell migration rate.

#### 4.1.7. Colony Formation Assay

To perform the colony formation assay, 1000 8305C cells were cultivated on six-well plates and exposed to BAY-293 treatment at concentrations of 1 μM, 10 μM, and 25 μM or solvent alone as a control. Following a 24 h treatment period, RPMI-1640 media supplemented with 10% FBS was added to the wells. After an incubation period of 10 days, the cells underwent two phosphate buffered saline (PBS) washes before being stained with 0.1% (*w*/*v*) crystal violet. Using a bright-field microscope, the stained cells were photographed (Zeiss, Oberkochen, Carl-Zeiss-Straße 22, Jena, Germany) [47].

#### 4.1.8. DNA Fragmentation Assay

Induction of apoptosis was analyzed based on detection of DNA fragmentation performed using agarose gel electrophoresis of the 8305C cells. Briefly 1.5 × 10^6^ cells were plated and treated with BAY-293 for 24 h. Then, the DNA was extracted using the REDExtract-N-Amp Tissue PCR Kit (XNAT-100RXN Sigma-Aldrich) according to the manufacturer’s instructions. Electrophoresis was performed for 30 min at 100 V with a 2% agarose gel. Then, the gels were photographed under ultraviolet light [48].

### 4.2. In Vivo Studies

#### 4.2.1. Animals

BALB/c nude male mice (25–30 g; 6–8 weeks of age) were procured from Envigo (Milan, Italy) for use in in vivo experiments. In a pathogen-free setting with 12 h of light and 12 h of darkness, the animals were housed in a controlled environment and supplied regular feed and water ad libitum.

#### 4.2.2. Orthotopic Model of ATC

In the orthotopic model of ATC, 3% isoflurane was used to anesthetize the BALB/c-nu/nu mice before 5 × 10^5^ 8305C cells, resuspended in 50 μL of saline, are injected into the right lobe of the thyroid using an insulin syringe with a 28G 1/2 needle. Following the operation, the animals were observed every day, and their overall health was evaluated by periodic weighing. After receiving the injection 13 days later, the mice were treated intraperitoneally with BAY-293 for 2 weeks, at two doses (10 and 50 mg/kg). The animals were sacrificed at the conclusion of the experiment, and the thyroids were removed, weighed, and examined [49].

The mice were randomly divided into four experimental groups, as described below:

1. SHAM group (8): intraperitoneal administration of saline;
2. ATC group (8): mice that received tumor cell inoculation intraperitoneally administered with saline;
3. ATC + BAY-293 10 mg/kg group (8): mice that received tumor cell inoculation intraperitoneally administered with BAY-293 at a dose of 10 mg/kg;
4. ATC + BAY-293 50 mg/kg (8): mice that received tumor cell inoculation intraperitoneally administered with BAY-293 at a dose of 50 mg/kg.

#### 4.2.3. Histological Evaluation

Hematoxylin and eosin staining for histological evaluation was performed as described above. Samples of thyroid tissue were promptly extracted using 10% buffered formalin. Following dehydration in xylene and graded ethanol, the samples were embedded in paraffin and sectioned at a thickness of 7 μm. Sections were examined under a Nikon Eclipse Ci-L optical microscope (Nikon Stroombaan, Amstelvee, The Netherlands) following hematoxylin and eosin staining. The histology findings are displayed at 20× (50 μm bar scale) and 40× (20 μm bar scale) magnifications. Every histological analysis was carried out in a blinded way.

#### 4.2.4. Masson’s Trichrome Staining

Using the Masson’s trichrome kit (Bio-Optica, Milan, Italy cat: 04-010802), morphological examination of the tumors was carried out on 5 µm sections following the manufacturer’s instructions. Using a Nikon Eclipse Ci-L microscope, the images were obtained at a magnification of 20× (50 μm bar scale) [50].

#### 4.2.5. Immunohistochemistry Assay

The expression of the tumor proliferation marker Ki-67 (1:100; sc-8426 Santa Cruz Biotechnology, Dallas, TX, USA) was evaluated by immunohistochemical analysis as previously described [51]. The images are shown at 20× magnification (50 μm of the bar scale) and 40× magnification (20 μm of the bar scale) using a Nikon Eclipse Ci-L.

#### 4.2.6. Western Blot Analysis for Thyroid Samples

Cytosolic proteins from thyroid sample were processed and sorted using electrophoresis and transferred to nitrocellulose membranes. After blocking the membranes with 5% (*w*/*v*) dried nonfat milk in buffered saline (PM) for 45 min at room temperature, the membranes were probed using the following specific antibodies: anti-PTEN (1:500; sc-7974; Santa Cruz Biotechnology), anti-SOS-1 (1:500; sc-17793; Santa Cruz Biotechnology), anti-p-p38 MAPK (1:500; sc-166182 Santa Cruz Biotechnology), anti-p-ERK (1:500; sc-7383; Santa Cruz Biotechnology), ERK (1:500; sc-514302; Santa Cruz Biotechnology), β-catenin (1:500; sc-7963; Santa Cruz Biotechnology), and anti-caspase 3 (1:500; sc-70498; Santa Cruz Biotechnology) in 1× PBS, 5% *w*/*v* dried nonfat milk, and 0.1% Tween-20 (PMT) at 4 °C overnight. Then, goat anti-mouse IgG secondary antibodies (1:2000, Jackson ImmunoResearch) or goat anti-rabbit IgG secondary antibodies (1:5000, Jackson ImmunoResearch) conjugated with peroxidase were incubated on membranes for 1 h at room temperature. As directed by the manufacturer (Thermo Fisher Scientific cat# 457), signals were detected using an enhanced chemiluminescence (ECL) detection system reagent. Protein bands indicating relative expression were measured using densitometry and the Bio-Rad ChemiDoc XRS + 6.1 software. The results were normalized to the levels of β-actin (sc-8432, 1:500; Santa Cruz Biotechnology), which served as an internal control.

#### 4.2.7. Statistical Analysis

The mean ± standard deviation (SD) of N observations is used to express all values. Three replicate samples were used for each analysis, which was carried out three times overall. One-way analysis of variance (ANOVA) was used to evaluate the data. For multiple comparisons, a Bonferroni post hoc test was performed. A *p*-value <  0.05 was deemed significant.

## 5. Conclusions

Through the inhibition of KRAS signaling, BAY-293 demonstrated the potential to disrupt aberrant cellular processes driven by oncogenic KRAS, such as proliferation, uncontrolled migration, survival, and metastasis. However, it is important to consider some limitations of the study. In particular, the cell lines used tend to lose the characteristics of the primary tumor, as they adapt to the in vitro growth conditions. Consequently, the use of these cell lines presents significant limitations. In recent years, greater impetus has been given to the study of human primary cell cultures, with a view to personalized cancer medicine [52]. Although further research is needed to define its clinical utility and potential as a safe and effective treatment option, BAY-293 has shown encouraging results for the treatment of ATC.

## Data Availability

All data in this study are included in this published article.

## Abbreviations

The following abbreviations are used in this manuscript:

ATC	Anaplastic thyroid carcinoma
PTC	Papillary thyroid carcinoma
FTC	Follicular thyroid carcinoma
TC	Thyroid carcinoma
GDP	Guanosine diphosphate
GTP	Guanosine triphosphate
PI3K	Phosphoinositide 3-kinase
MAPK	Mitogen-activated protein kinase
SOS-1	Son of sevenless 1
MTT	3-(4,5-Dimethylthiazol-2-yl)-2,5-diphenyltetrazolium bromide
PTEN	Phosphatase and tensin homolog
p38	Phosphorylated p38 mitogen-activated protein kinase
ERK	Extracellular signal-regulated kinase
Bcl2	B-cell leukemia/lymphoma 2 protein
BAX	Bcl2-associated X, apoptosis regulator
p53	Tumor protein p53
